# Supplementary material for: Distinct stabilization of the human T cell leukemia virus type 1 immature Gag lattice
Source: Nat Struct Mol Biol. 2024 Sep 6;32(2):268–76. doi: 10.1038/s41594-024-01390-8 (PMC11832423; doi:10.1038/s41594-024-01390-8)
Supplement: Supplementary file 1 — Supplementary Fig. 1 and Tables 1 and 2. [file 41594_2024_1390_MOESM1_ESM.pdf]

nature structural & molecular biology

Supplementary information https://doi.org/10.1038/s41594-024-01390-8

Distinct stabilization of the human T cell leukemia virus type 1 immature Gag lattice

In the format provided by the authors and unedited

Supplementary Figures

Uncropped blots for Extended Data Figure 7a

Cell culture supernatant

Cell lysate

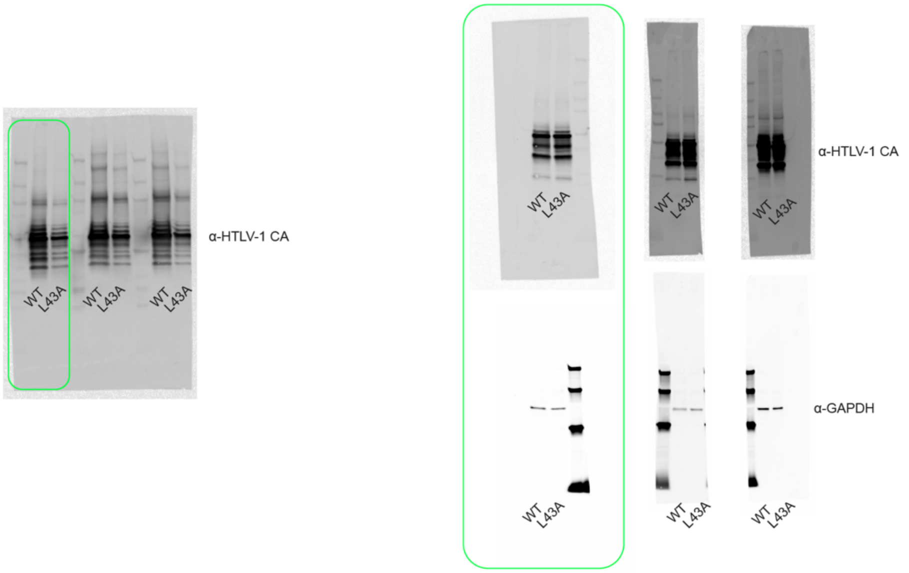

Uncropped blots for Extended Data Figure 7c

Cell culture supernatant

Cell lysate

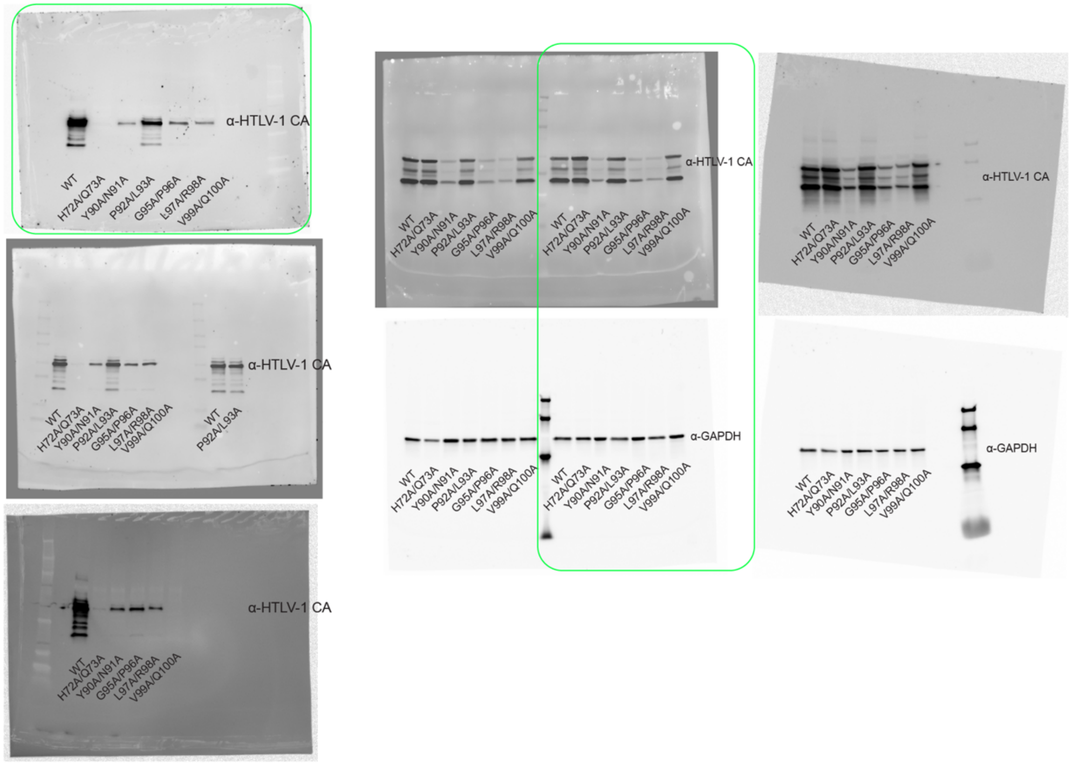

Supplementary Figure 1: Uncropped Western blots for data shown in Extended Data Figure 7
The representative blots shown in Extended Data Figure 7 are highlighted with green boundaries. Given that the cell culture supernatant contains only released virus-like particles and no loading control, the cell culture supernatant data was measured against the cell lysate data.

Supplementary Tables

Extended Data Figure 7a: cell culture supernatant

	L43A
***P* value**	<0.0001
**Average ± SEM (in %)**	40.31 ± 2.338
**95% Confidence Interval**	30.25 to 50.37
**n**	3

Extended Data Figure 7a: cell lysate

	L43A
***P* value**	0,1267
**Average ± SEM (in %)**	124.9 ± 12.93
**95% Confidence Interval**	69.24 to 180.52
**n**	3

Extended Data Figure 7c: cell culture supernatant

	H72A/Q73A	Y90A/N91A	P92A/L93A	G95A/P96A	L97A/R98A	V99A/Q100A
***P* value**	<0.0001	<0.0001	0,0006	<0.0001	<0.0001	<0.0001
**Average ± SEM (in %)**	0.5240 ± 0.2660	6.986 ± 1.930	42.56 ± 5.871	10.93 ± 1.724	7.920 ± 2.232	0.00033 ± 0.00033
**95% Confidence Interval**	-0.62 to 1.67	-1.32 to 15.29	17.29 to 67.82	3.52 to 18.35	-1.68 to 17.52	-0.0011 to 0.0018
**n**	3	3	3	3	3	3

Extended Data Figure 7c: cell lysate

	H72A/Q73A	Y90A/N91A	P92A/L93A	G95A/P96A	L97A/R98A	V99A/Q100A
***P* value**	0,0727	<0.0001	0,3729	<0.0001	<0.0001	0,0008
**Average ± SEM (in %)**	124.2 ± 7.393	14.45 ± 0.6167	89.05 ± 6.789	16.74 ± 1.437	11.43 ± 1.081	64.40 ± 3.077
**95% Confidence Interval**	109.71 to 138.69	13.24 to 15.65	75.74 to 102.36	13.92 to 19.55	9.31 to 13.54	58.36 to 70.43
**n**	3	3	3	3	3	3

Supplementary Table 1: Statistical analysis values for Extended Data Figure 7a,c
Average ± SEM is normalized to WT. An average value of 100 indicates that mutant production is equivalent to WT. The 95% confidence interval is expressed as a percentage relative to WT.

	HTLV-1			HIV-1		
	CA	CA-NTD	CA-CTD	CA	CA-NTD	CA-CTD
Pre-assembly Tm [°C]	51.83	55.61	57.96	47.25	49.86	63.62
Post-assembly Tm [°C]	47.09	48.95	57.25	44.63	46.36	62.66
Pre-assembly Turbidity IP [°C]	48.05	55.35	-	47.64	50.24	-
Post-assembly Turbidity IP [°C]	44.97	46.27	-	44.06	45.90	-

Supplementary Table 2: Melting temperatures (Tm) and Turbidity inflection points (IPs) of HTLV-1 and HIV-1 CA constructs compared in pre-assembly and post-assembly conditions.
