# Supplementary figures and images for: Distinct stabilization of the human T cell leukemia virus type 1 immature Gag lattice
Source: Nat Struct Mol Biol. 2024 Sep 6;32(2):268–76. doi: 10.1038/s41594-024-01390-8 (PMC11832423; doi:10.1038/s41594-024-01390-8)

Uncropped gel for Extended Data Figure 4a

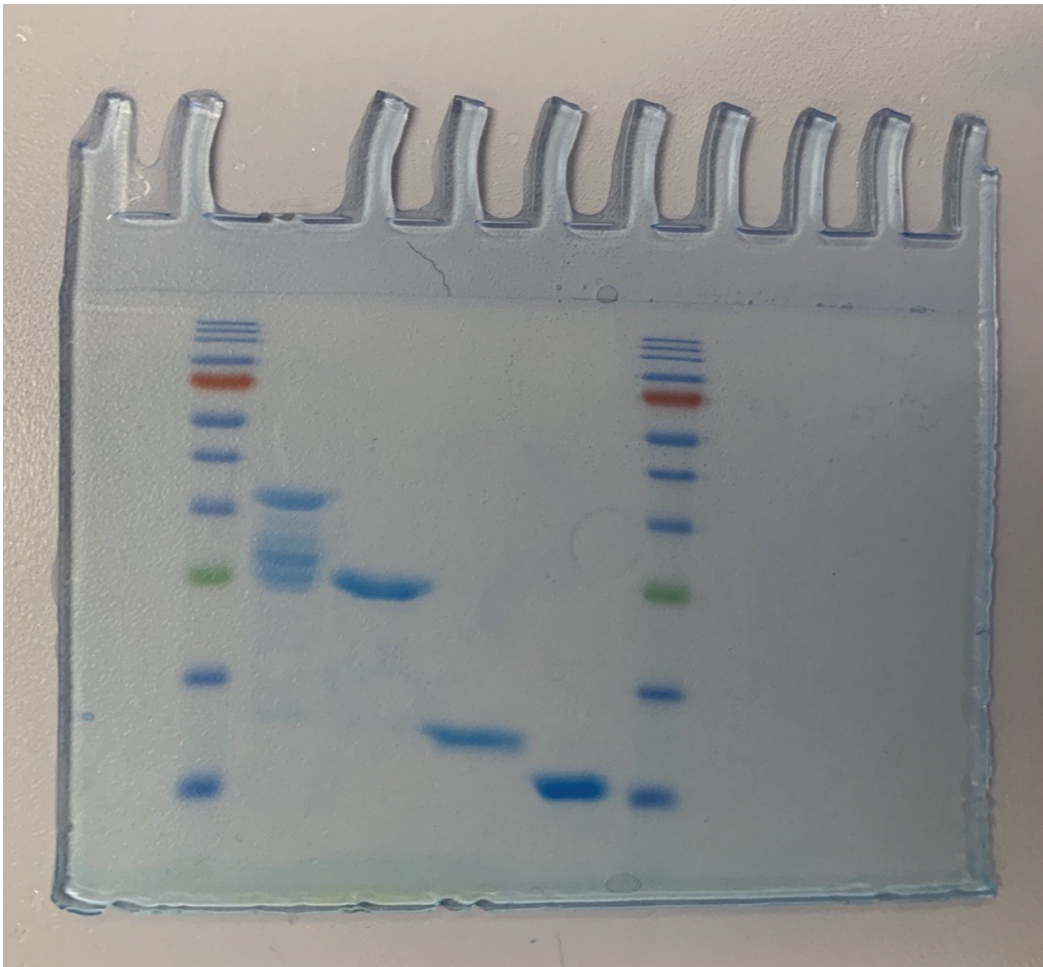

Uncropped gel for Extended Data Figure 4c

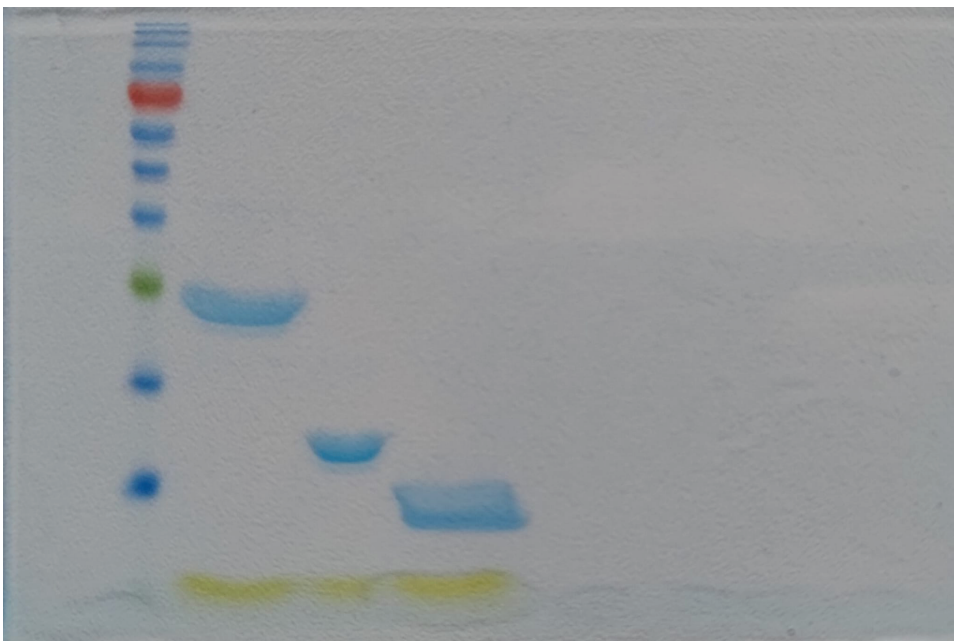

Supplement: Supplementary file 7 — Uncropped, nonannotated gels. [file 41594_2024_1390_MOESM7_ESM.pdf]
