# Supplementary material for: Distinct stabilization of the human T cell leukemia virus type 1 immature Gag lattice
Source: Nat Struct Mol Biol. 2024 Sep 6;32(2):268–76. doi: 10.1038/s41594-024-01390-8 (PMC11832423; doi:10.1038/s41594-024-01390-8)

<u>Uncropped blots for Extended Data Figure 7A</u>

Cell culture supernatant

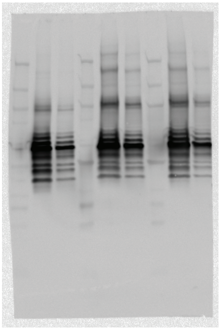

Cell lysate

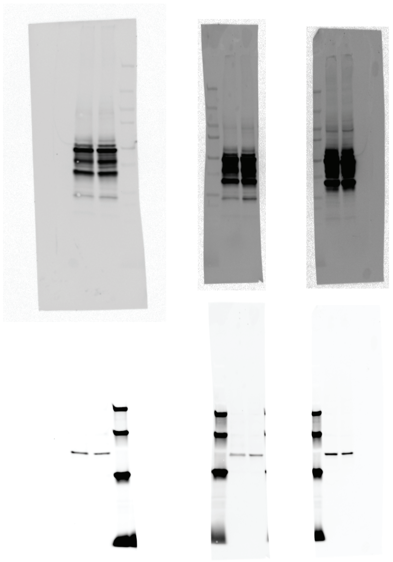

<u>Uncropped blots for Extended Data Figure 7C</u>

Cell culture supernatant

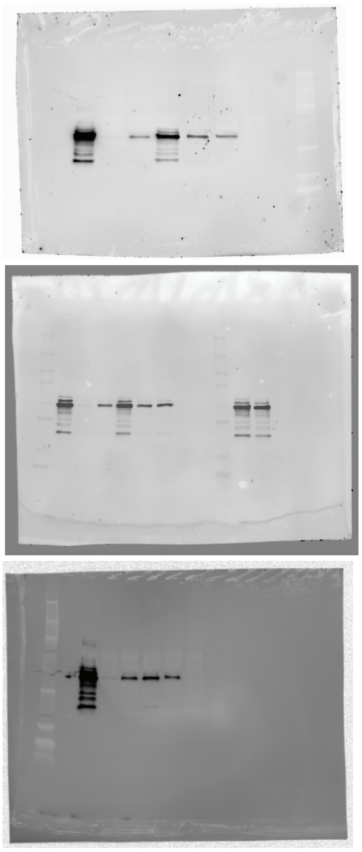

Cell lysate

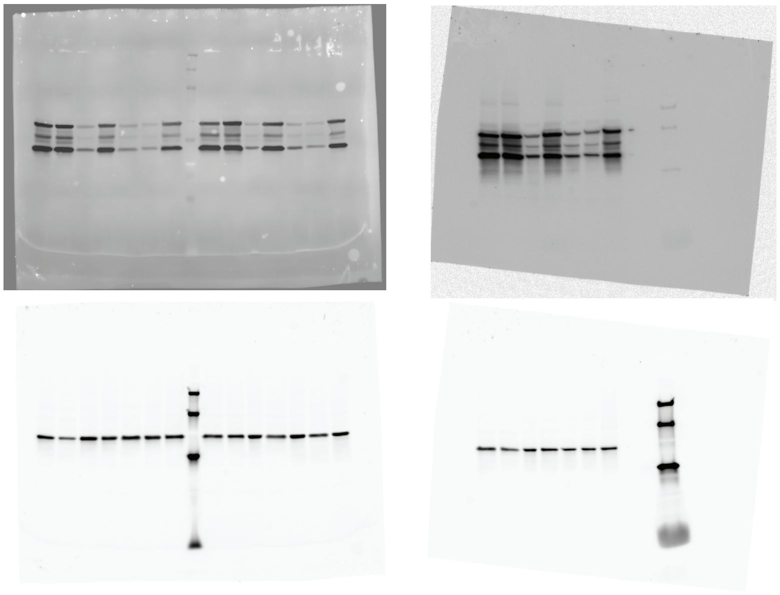

Supplement: Supplementary file 8 — Uncropped, nonannotated blots. [file 41594_2024_1390_MOESM8_ESM.pdf]
